# Transcriptomic analysis of common carp anterior kidney during *Cyprinid herpesvirus 3* infection: Immunoglobulin repertoire and homologue functional divergence

**DOI:** 10.1038/srep41531

**Published:** 2017-02-02

**Authors:** Matthew J. Neave, Agus Sunarto, Kenneth A. McColl

**Affiliations:** 1 CSIRO Health and Biosecurity, Australian Animal Health Laboratory, Geelong, VIC 3220, Australia; 2 AMAFRAD Centre for Fisheries Research and Development, Fish Health Research Laboratory, Jakarta 12540, Indonesia

## Abstract

*Cyprinid herpesvirus* 3 (CyHV-3) infects koi and common carp and causes widespread mortalities. While the virus is a significant concern for aquaculture operations in many countries, in Australia the virus may be a useful biocontrol agent for pest carp. However, carp immune responses to CyHV-3, and the molecular mechanisms underpinning resistance, are not well understood. Here we used RNA-Seq on carp during different phases of CyHV-3 infection to detect the gene expression dynamics of both host and virus simultaneously. During acute CyHV-3 infection, the carp host modified the expression of genes involved in various immune systems and detoxification pathways. Moreover, the activated pathways were skewed toward humoral immune responses, which may have been influenced by the virus itself. Many immune-related genes were duplicated in the carp genome, and often these were expressed differently across the infection phases. Of particular interest were two interleukin-10 homologues that were not expressed synchronously, suggesting neo- or sub-functionalization. The carp immunoglobulin repertoire significantly diversified during active CyHV-3 infection, which was followed by the selection of high-affinity B-cells. This is indicative of a developing adaptive immune response, and is the first attempt to use RNA-Seq to understand this process in fish during a viral infection.

*Cyprinid herpesvirus* 3 (CyHV-3) is a large double-stranded DNA virus^1^ that was first recognized in the late 1990s^2^,^3^. The virus specifically infects koi and common carp (*Cyprinus carpio*) and causes widespread mortalities. Initial outbreaks of CyHV-3, for example, can result in population losses of 80–95%^4^,^5^. This is particularly concerning for the carp aquaculture industry, where CyHV-3 outbreaks result in substantial economic losses^6^. For example, an Indonesian outbreak in 2002 and 2003 is estimated to have cost farmers up to US$15 million^5^. CyHV-3 is thought to cause mortality by disrupting the osmoregulatory function of the host^7^, and by weakening the host immune system, thereby leaving the host more susceptible to infection by other viruses, bacteria or fungi^8^.

CyHV-3 belongs to the *Alloherpesviridae* family within the order *Herpesvirales*^1^. It has a genome size of 295 kb, which contains 156 putative open reading frames (ORFs), including two 22 kb terminal repeats^9^. CyHV-3 gene transcription follows a co-ordinated and sequential pattern during active host infection, similar to other herpesviruses^10^,^11^. Accordingly, the ORFs can be divided into 3 broad groups reflecting these events: 15 immediate-early; 112 early; 22 late; 7 remain unclassified^10^. Although the functional role of many CyHV-3 proteins is unclear, the characterised ORFs include various kinases, RING gene families, membrane glycoproteins and structural virion components, as well as pseudogenes and frameshifted ORFs^1^,^8^,^9^. A particularly interesting group of ORFs seems to have evolved to evade the host’s immune response. These include a functional Interleukin-10 (IL-10), known as khvIL-10, which is homologous to the carp’s own IL-10 and may mediate an immunosuppression mechanism^12^,^13^,^14^. Moreover, ORF12 and ORF4 are viral homologues of tumor necrosis factor receptors (TNFRs), which are often used by large DNA viruses to regulate host immune responses^15^,^16^.

Interestingly, CyHV-3 may be useful in some countries as a biocontrol agent. In Australia, for example, carp are an introduced invasive species that cause major disruptions to native ecosystems^17^,^18^. Indeed, carp are now the most abundant freshwater fish in south-east Australia, and in some instances comprise 90% of all fish biomass^19^. This results in the dislocation of native species, increased water turbidity, the loss of aquatic vegetation, and alterations in zooplankton and benthic invertebrate diversity^18^. Several characteristics of CyHV-3 suggest that it could be a suitable biocontrol agent for carp, including its lethality, specificity (only infects target species), and its rapid spread through populations^8^,^17^.

One aspect that needs further examination, however, is the development of the carp immune response to CyHV-3 infection, and subsequent mechanisms of carp resistance. A thorough understanding of carp immune responses and resistance mechanisms is required for controlling viral spread in aquaculture through the development of both CyHV-3 resistant carp and vaccines, and conversely, for facilitating viral spread in biocontrol programs by ensuring continued viral virulence in pest populations. Moreover, immunological responses can reveal if carp have been previously exposed to CyHV-3 and if they are potential carriers that may spread CyHV-3 to naïve fish.

Carp that survive a primary infection with CyHV-3 have superior resistance to subsequent infections, and mortalities are significantly reduced^20^,^21^. These fish may have greater innate resistance through pre-existing genetic factors, or alternatively, they may have mounted an effective adaptive immune response. Innate resistance of carp to CyHV-3 has been linked to single nucleotide polymorphisms (SNPs) in IL-10 genes^22^, specific genotypes of major histocompatibility class II genes^23^, and higher regulation of various immune factors^24^. In terms of adaptive immunity, carp have been shown to mount a cell-mediated immune response^25^, and to produce specific immunoglobulin (Ig) molecules in response to CyHV-3^21^,^26^. Most studies to date have used serological methods to analyse the carp immune response, which can be challenging because immunological systems in fish are more primitive compared to mammals. For example, there are fewer Ig classes, no class switching, and the process of affinity maturation is much less efficient^27^.

Here we used RNA-Seq to simultaneously examine the gene expression dynamics of CyHV-3 and carp during various infection phases. Apart from identifying components of the cell-mediated immune response, a molecular approach, rather than serological, may also improve sensitivity for the detection of specific anti-CyHV-3 Ig. Our aims were to improve the current understanding of CyHV-3 genome transcription, carp resistance and acquired immunity, and to improve early detection of carp that have been exposed to CyHV-3. This would have implications for both aquaculture and biocontrol programs. Three different phases of infection were used to explore immune development in carp, including acutely infected fish (acute phase), infected fish held at a low, non-permissive temperature for CyHV-3 replication (persistent phase), and infected fish reacclimatized to a permissive temperature after being held at the non-permissive temperature (reactivation phase). The persistent phase was not intended to mimic a latent infection, but rather a low-level persistent infection that could then be stimulated in the reactivation phase. We hypothesized that carp in the acute phase would show an early immune response, with some increase in Ig diversity. Infected carp in the persistent phase were expected to slowly develop a mature immune response in the face of suppressed CyHV-3 replication. Finally, carp in the reactivation phase were predicted to develop a stronger immune response due to reactivated virus replication, and greater Ig diversity with developing specificity, due to their previously acquired immunity.

## Results

### Genome mapping and replicate similarity

Carp immune responses and CyHV-3 open reading frame (ORF) expression were examined during three phases of infection: acute, persistent and reactivation. Fish from each of these phases were sampled, messenger RNA (mRNA) was extracted and RNA-Seq technology was used to produce several million high quality reads per sample (Table 1). After cleaning, a large proportion of the reads were successfully mapped to the carp genome, with good coverage for gene expression analyses (Table 1). The percentage of CyHV-3 reads in carp cells was highest in the acute and reactivated infections, as expected, although there was variability between the replicates (Table 1). For example, acute replicate 1 contained ~1.44% viral reads compared to ~0.02% in both other replicates. In the reactivated samples, replicate 3 had ~0.03% CyHV-3 reads, whereas the other replicates had an order of magnitude fewer. In both persistent and mock samples, low numbers of reads mapped to the CyHV-3 genome. The few CyHV-3 reads detected in the mock replicates may be the result of low-level cross-contamination, which is common in high-throughput sequencing studies.

### Gene expression determined by RNA-Seq and qRT-PCR are highly correlated

To validate our approach, we compared the RNA-Seq expression levels from several representative CyHV-3 ORFs to expression levels determined by quantitative reverse transcription-PCR (qRT-PCR) using the same samples. Six ORFs that span the CyHV-3 genome and are transcribed at different stages of active infection were chosen for the comparison (Supplementary Fig. S1). ORF expression values determined by RNA-Seq and qRT-PCR were significantly correlated and had a Pearson’s r value greater than 0.90 for five of the six ORFs tested (Supplementary Fig. S1). The only exception was ORF78, which had a slightly lower correlation (Pearson’s r = 0.86, p = 0.059). These results suggest that RNA-Seq is as sensitive and accurate as qRT-PCR for determining viral gene expression *in vivo*, with the added benefit of characterizing expression over the entire genome. This means that not only can all viral ORFs be examined, but other genomic regions that may not be annotated easily, such as micro-RNAs and other non-coding RNAs, can also be studied.

### CyHV-3 gene expression follows a co-ordinated pattern across infection phases

**Figure 1:**
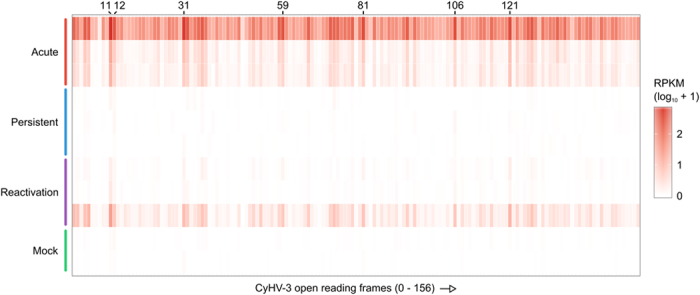
Normalized gene expression from CyHV-3 open reading frames (ORFs). Values were normalised to reads per kilobase of transcript per million mapped reads (RPKM) and log_10_ transformed to enable sample comparison.

Although absolute expression levels of CyHV-3 were much higher in acute replicate 1, the relative expression pattern was similar to the other acute replicates and to the reactivated samples (Fig. 1). For example, the most highly expressed ORFs were the same within each sample (Fig. 1). These included ORF11 (unknown function), ORF12 (tumor necrosis factor receptor), ORF31 (similar to eukaryotic PLAC8 proteins), ORF59 (predicted transmembrane protein), ORF81 (viral envelope, type III membrane protein with 4 transmembrane domains), ORF106 (predicted transmembrane protein), and ORF121 (function unknown)^1^,^9^. Interestingly, the largest peak in the raw coverage profiles was consistently located within the 22 kb terminal repeat region of the CyHV-3 genome (Supplementary Fig. S2). This peak was between ORF5 and ORF6, outside both protein coding regions, and appeared to be associated with the polyadenylation sites for each gene^9^.

To determine if the most highly expressed CyHV-3 ORFs were indeed the same in the different infection phases, and if any ORFs were significantly differently expressed, pairwise comparisons were conducted (Supplementary Fig. S3). Comparison of the acute and reactivation phases showed an approximately linear relationship, confirming the coordinated expression of ORFs in the CyHV-3 genome (Supplementary Fig. S3). CyHV-3 ORFs in the acute phase were clearly more highly expressed than in the other infection phases, and many of the expression values were significantly different. Several of the acute expression values were also found to be non-significant, despite appearing to have much higher levels. This is likely due to variability in the acute replicates (replicate 1 had far more CyHV-3 reads), reducing the power of the test to find significant differences.

### CyHV-3 infection extensively modifies gene expression dynamics in carp

**Figure 2:**
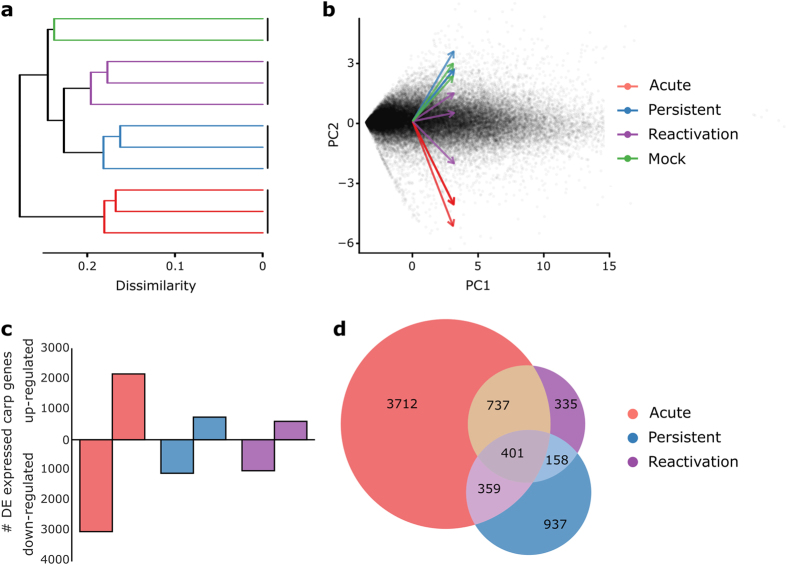
Similarities in carp gene expression profiles using Jensen-Shannon distances (**a**) and principal components (**b**), and significantly differently expressed carp genes in each phase (**c**) and common to different phases (**d**). In each plot the colours indicate the phase of infection. The numbers in (**d**) indicate significantly differently expressed genes in each phase and the overlapping segments indicate the number of genes differentially expressed in, and between, phases of infection. PC is an acronym for principal component.

Replicate carp samples within each phase had similar gene expression profiles, providing high power for significance testing (Fig. 2a,b). Interestingly, the carp gene expression profiles changed in an approximately linear fashion from phases without active CyHV-3 replication, to reactivated samples, to samples in the acute infection phase (i.e., principal component 2 progressing from positive to negative values in Fig. 2b). Moreover, several hundred to several thousand carp genes were significantly up- or down-regulated in response to the CyHV-3 infection phases (Fig. 2c). The highest number of differentially expressed genes was seen in the acute phase, and more genes were down-regulated than up-regulated (Fig. 2c). A high proportion of differentially expressed genes in the reactivation phase were also differentially expressed in the acute phase, while in contrast, differentially expressed genes in the persistent phase were often unique to that group (Fig. 1d). The top differentially expressed genes in the acute phase were related to the detoxification of foreign substrates and immune responses, including nitric oxide synthase, microfibril-associated glycoprotein 4, cytoglobin 1, myeloperoxidase, glutathione S-transferase 3, a signal peptide and immune-responsive gene 1 (Table 2). In the persistent and reactivation phases, significant expression differences were also seen for immune related and detoxification related genes, although often they were down-regulated. These included single Ig IL-1-related receptor, cytosolic sulfotransferase, signal peptides, trans-1,2-dihydrobenzene-1,2-diol dehydrogenase, CXC chemokines and glutathione S-transferase 3 (Table 2).

### Carp humoral immune response predominates in acute CyHV-3 infections

**Figure 3:**
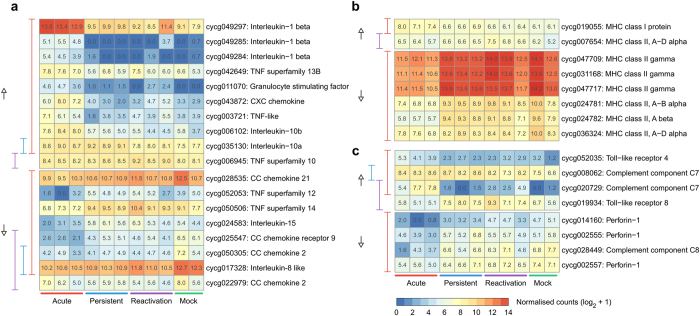
Significantly differentially expressed carp genes that are annotated with the gene ontology term “immune response” (GO:0006955). The genes have been further categorized into cytokines (**a**), major histocompatibility classes (**b**) or other immune system components (**c**). For better visualisation, raw counts have been normalised (log_2_ + 1). The bars on the left side of each heatmap indicate which genes were significantly differentially expressed in each infection phase (red = acute, blue = persistent, violet = reactivation) compared to the mock controls, and the arrows indicate an up- or down-regulation. TNF is an acronym for tumor necrosis factor.

The immune responses of carp infected with CyHV-3 were examined more closely by analysing differentially expressed genes annotated with the gene ontology (GO) term “immune response” (GO:0006955; Fig. 3). When fish in the acute phase were compared to mock infected fish, several cytokine molecules were up-regulated, including interleukin-1 beta (IL-1b) homologues, interleukin-10 (IL-10) homologues, granulocyte stimulating factor, various chemokines and some, but not all, tumor necrosis factors (TNF) (Fig. 3a). On the other hand, interleukin-15 (Fig. 3a) and almost all major histocompatibility class II (MHC II) antigens were down-regulated during acute CyHV-3 infection, while one MHC class I antigen was up-regulated (Fig. 3b). Moreover, C7 components of the complement cascade were up-regulated, and various perforin genes, which are associated with cytotoxic T cells in a cell-mediated immune response, were down-regulated in the acute phase compared to the controls (Fig. 3c). The up-regulation of IL-10 and at least one component of the complement system, plus the blanket down-regulation of MHC II antigens and perforin molecules, suggests that carp in the acute phase of CyHV-3 infection predominantly mount a T Helper 2 (Th2) response, which stimulates the humoral immune system at the expense of a cell-mediated immune response.

In contrast, fish in the persistent and reactivation phases altered the expression of far fewer immune-related genes. For example, they did not alter the expression of IL-1b homologues, granulocyte stimulating factor or most TNF transcripts, compared to mock infected fish (Fig. 3a). Moreover, MHC I and II antigens in both the persistent and reactivation phases were expressed in similar levels to the mock controls (Fig. 3b). In the persistent phase, however, an IL-10 homologue (IL-10a; cycg035130) was up-regulated compared to the controls, while another was unchanged (IL-10b; cycg0006102) (Fig. 3a). These differences in IL-10 homologue expression across CyHV-3 infection phases have interesting implications for this important anti-inflammatory cytokine and these were investigated further.

### Differential interleukin-10 homologue expression

**Figure 4:**
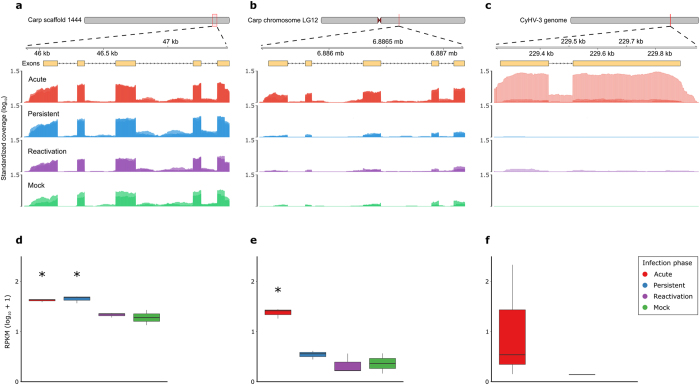
Genomic location, gene splicing and expression differences for interleukin-10 homologues in carp, IL-10a (cycg035130; **a**,**d**) and IL-10b (cycg006102; **b**,**e**), and the CyHV-3 homologue (**c**,**f**). Exons are indicated by yellow boxes and standardized coverage is the depth per million total mapped reads in each sample library (log_10_ scale; **a,b,c**). Reads per kilobase per million mapped reads (RPKM) in d, e and f indicate the combined expression signal over the whole gene and are normalized for library and gene size. Asterisks indicate a significant difference compared to the mock infected fish (**d,e**).

We analysed the carp genome for the presence of duplicated immune genes using reciprocal best match BLAST searches, and found numerous duplicated genes involved in a wide range of different immune pathways (Supplementary Fig. S4). Although the expression of duplicated genes was moderately correlated (Supplementary Fig. S4, inset), many gene pairs were differentially expressed during CyHV-3 infection (Supplementary Fig. S4). These included major histocompatibility antigens, tumor necrosis factors and various cytokine molecules (Supplementary Fig. S4). Of particular interest were expression changes in IL-10, which is an important modulator of immune responses and is frequently analysed in other carp experiments^13^,^22^,^28^. In the carp genome, two copies of IL-10, designated IL-10a (cycg035130; Fig. 4a,c) and IL-10b (cycg006102; Fig. 4b,d), are present^22^,^29^. These homologues were detected in the carp genome assembly^30^, and were also confirmed by PCR amplification and Sanger sequencing (GenBank accessions KX964677 and KX964678). The two IL-10 homologues have high amino acid identity (84%) and similar gene splicing organization, with each containing 5 exons (Fig. 4a,b). Moreover, the expression of the exons was highly conserved across the two genes (Fig. 4a,b). Despite the high sequence conservation and splicing similarity, however, the homologues were expressed differently during different phases of infection. For example, IL-10b was only significantly up-regulated in the acute phase (Fig. 4e), while IL-10a was up-regulated in both the acute and persistent phases and had a greater baseline expression level (Fig. 4d). The CyHV-3 genome also contains a captured homologue of carp IL-10, which shares 24% and 25% amino acid identity with carp IL-10a and IL-10b, respectively (Fig. 4c,f). Significantly, in acute replicate 1, viral IL-10 was expressed at more than double the rate of the carp’s own IL-10 homologues (Fig. 4).

### CyHV-3 infection increases carp immunoglobulin repertoire and V-region selection pressure

**Figure 5:**
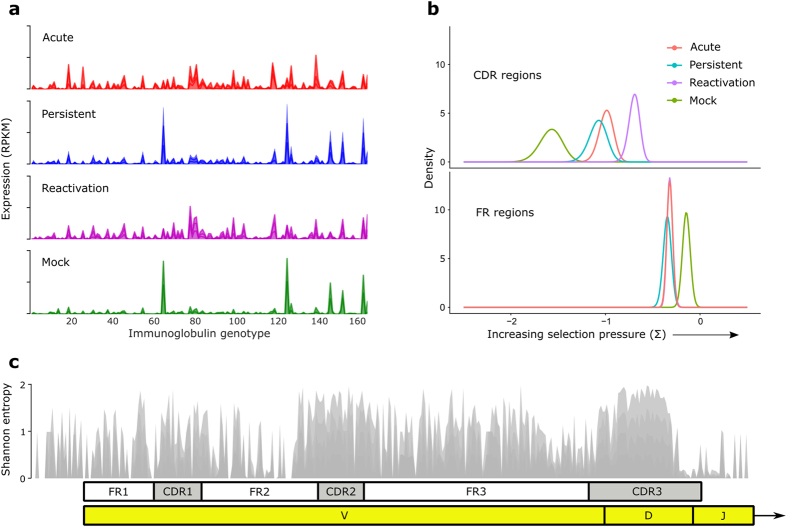
Carp immunoglobulin (Ig) repertoire in different phases of infection (**a**), selection pressure on complementarity determining regions (CDR) and framework regions (FR) of carp Ig molecules (**b**), and nucleotide diversity (as measured by Shannon entropy) in a multiple alignment of carp Ig transcripts (**c**). Selection pressure in (**b**) was estimated using somatic mutation patterns and the Bayesian estimation of Antigen-driven SELectIoN (BASELINe) algorithm (Yaari *et al*. 2012). Variable (V), diversity (D) and joining (J) regions of the Ig transcript in (**c**) are annotated.

The developing immune response of carp in response to CyHV-3 infection was further scrutinized by examining the immunoglobulin (Ig) repertoire in each phase of infection (Fig. 5). In the absence of infection (mock) or in the face of a low level active infection (persistent phase), carp expressed remarkably similar Ig genotypes with similar expression levels (Fig. 5a). In contrast, fish in the acute and reactivation phases expressed a significantly greater diversity of Ig genotypes (p < 0.05), and did not highly express the mock Ig genotypes (Fig. 5a). Moreover, somatic mutation patterns revealed that Ig transcripts from the acute and reactivation groups were under higher selection pressure in complementarity determining regions (CDR) and lower selection pressure in framework regions (FR). This pattern was reversed for Ig transcripts in mock fish (Fig. 5b). In fact, fish that were subjected to the longest periods of infection with CyHV-3, i.e., in the reactivation phase, had the highest overall selection pressure in CDR regions (Fig. 5b). A global alignment of all carp Ig transcripts indicated that the highest nucleotide diversity was located within CDRs, particularly CDR3 (Fig. 5c), confirming that these regions are an important source of diversity for antigen matching.

## Discussion

We used RNA-Seq on carp infected with CyHV-3 to analyse genome-wide expression changes in both the virus and host, including CyHV-3 transcription patterns, carp immune responses, expression differences in immune-related homologues, and carp Ig diversity. Three different phases of CyHV-3 infection were used to gain a more comprehensive understanding of the interplay between virus and host, including acute (initial severe infection), persistent (infected but little or no CyHV-3 replication) and reactivation (active infection following persistent phase).

CyHV-3 ORFs were consistently expressed in the same relative proportions across the sample replicates and infection phases, suggesting a sequential and coordinated expression pattern, as previously reported^10^. A number of the highly expressed viral ORFs have eukaryotic counterparts, suggesting a role in evasion of the host immune response. These include ORF31, which has homology to eukaryotic PLAC8 proteins, and ORF12, a viral homologue of tumor necrosis factor receptor (TNFR) SF1^1^,^15^. Tumor necrosis factors (TNFs) are inflammatory cytokines that induce several immunological responses, including apoptosis of infected cells^31^. Virus-encoded TNFRs counteract TNF activity of the host by functioning as decoy receptors^32^. Through this act of mimicry the virus can modulate the host’s immune response and ensure its own survival^15^,^16^. In concordance with the results here, CyHV-3 TNFR was found to be one of the most abundant proteins in cell culture^33^, suggesting that this is a major immune avoidance mechanism for the virus. A disproportionately large number of the highly expressed CyHV-3 ORFs contained transmembrane domains, demonstrating the importance of signalling and cross membrane molecule transport to CyHV-3 functioning. ORF81, which is part of the viral envelope, is a type III membrane protein with 4 transmembrane domains and was the first CyHV-3 protein to be functionally characterised^34^. This protein was found to be abundant in CyHV-3 cell culture, supporting the high expression results seen here, and has been recombined into a plasmid for use in a vaccine experiment due to its high immunogenicity^35^. The high abundance and immunogenicity of ORF81 makes this an important genomic region in the study of viral-host co-evolution, particularly for studies examining the development of host resistance and the feasibility of CyHV-3 as a biocontrol agent for carp.

In the carp host, the greatest number of gene expression changes were seen in the acute phase of CyHV-3 infection, with over 5,000 genes significantly up- or down-regulated compared to mock infected fish. Interestingly, fewer gene expression changes were seen for fish in the reactivation phase (approximately 1,600 genes up- or down-regulated) despite these fish also experiencing a severe infection. This discrepancy may have arisen because fish in the reactivation phase had been pre-exposed to CyHV-3, possibly leading to the development of a more focused immune response. This is consistent with other studies, which show that following initial expression changes, many genes return to control levels after several days of infection^13^,^24^,^36^. In another study that used high-throughput sequencing to assemble a carp transcriptome, more than 20,000 differentially expressed genes were detected during CyHV-3 infection^37^. This is significantly more than detected here, which probably relates to the different bioinformatics techniques used, i.e., transcriptome assembly versus genome mapping, and different experimental designs. Interestingly however, they also found that more carp genes were down-regulated than up-regulated in response to CyHV-3, which likely reflects the suppression of broad metabolic pathways in favour of a targeted immune response.

Many of the most differentially expressed carp genes in this experiment have also been detected in previous reports. For example, nitric oxide synthase (NOS), which was highly expressed in the kidney of infected carp here, is also highly expressed in carp skin, intestine, spleen and gills during CyHV-3 infection^36^,^38^. The up-regulation of NOS can control the replication of many viruses through inhibition of viral protease activity^39^, and may be a general host defence mechanism. Microfibril-associated glycoprotein 4 (MFAP4) was also very highly expressed in carp in the acute phase of CyHV-3 infection. Interestingly, Rakus *et al*. found that MFAP4 was more highly expressed in carp with greater resistance to CyHV-3, indicating its importance to carp immunity^24^. Indeed, Niu *et al*. suggested that MFAP4 is the teleost homologue of ficolins, which can recognize pathogens, activate the complement system, initiate opsonisation, and trigger other pathogen clearing mechanisms^40^. The up-regulation of both myeloperoxidase (MPO) and cytoglobin during active CyHV-3 infection may be interrelated. For example, MPO is expressed by granulocytes to catalyse the production of acids and kill pathogens^41^, however, this can also cause oxidative damage to host cells, which in turn, may stimulate the production of cytoglobin as a protective measure^42^. Similarly, glutathione S-transferase (GST) is a well-known antioxidant enzyme^43^. In this study, however, it was down-regulated in response to CyHV-3 infection. This may be related to the many roles of GSTs, which include the suppression of apoptosis by inhibition of c-Jun NH_2_-terminal kinase (JNK) and p38^44^. Thus, the down-regulation of GST in acutely infected fish would allow apoptosis of infected cells. Overall, carp infected with CyHV-3 alter the expression of a broad group of genes involved in general immune responses, clearing pathogens and dealing with oxidative stress induced by the virus directly, or as a consequence of other detoxification pathways.

An examination of specific adaptive immune responses in carp revealed differences between acutely infected carp, and those in the persistent and reactivation groups. Specifically, fish in the acute phase up-regulated interleukin-1 beta (IL-1b) homologues, interleukin-10 (IL-10) homologues and components of the complement system, while most major histocompatibility class II (MHC II) antigens and perforin molecules were down-regulated. This suggests that a humoral, as opposed to cell-mediated, immune response was mounted by carp in the late stages of acute CyHV-3 infection. For example, an increase in IL-10 expression can down-regulate cytokines involved in T Helper 1 (Th1) responses, MHC II antigens and other macrophage stimulatory molecules (cell-mediated responses), in addition to catalysing the proliferation of B-cells and antibody production (humoral responses)^45^. Indeed, in the current study, greater IL-10 expression in infected carp corresponded with decreases in MHC II antigen and perforin expression. Moreover, several components of the complement cascade, which can help clear immune complexes and pathogens in a humoral response^46^, were up-regulated in acutely infected carp. This predominance of humoral immune responses may be unexpected during the latter stages of CyHV-3 infection, where CyHV-3 particles have presumably become established within cells. Interestingly, the type of immune response mounted by carp might be influenced by the virus itself. For example, CyHV-3 encodes a functional IL-10 homologue^12^,^14^,^33^,^47^, which in one acute replicate was expressed at double the rate of the carp’s own IL-10 homologues. This would artificially increase local or systemic IL-10 concentrations, further encouraging a humoral immune response and suppression of cell-mediated immunity, thus allowing intracellular virus to survive and to potentially develop latency. On the other hand, carp in the persistent and reactivation phases expressed a range of genes that suggested a balance between humoral and cell-mediated immune responses. This divergence may be attributed to longer periods of infection in the persistent and reactivation phases, allowing for a more targeted and mature response.

The carp genome has been studied with particular interest due to a relatively recent whole genome duplication (WGD) event (~8.2 million years ago), which allows researchers to examine the initial consequences of genome duplication on gene function^30^,^48^,^49^,^50^. For example, WGD often produces extensive genetic redundancy, which results in gene deletions or functional divergence. Interestingly however, Li *et al*. found that recently duplicated carp genes have mostly been retained, although approximately half of the duplicates were not expressed synchronously, suggesting functional divergence or sub-functionalization^49^. In this study, we found that many immune-related genes from a broad group of pathways were also duplicated. Although the expression of duplicated genes was moderately correlated, a large number of homologues showed significant dissociation of expression, including genes that are central immune components, such as tumor necrosis factors, interleukins and various other cytokines. This has important implications for understanding carp immune responses.

One such homologue pair was IL-10a and IL-10b, which showed particularly large expression differences. Specifically, both homologues were up-regulated during the acute infection phase, but only IL-10a was up-regulated during the persistent phase. In addition, IL-10a had a higher baseline expression level in mock infected carp and during the persistent phase compared to IL-10b. This suggests either sub-functionalization (both homologues retain some functionality), neo-functionalization (new homologue functionality), or that the homologues are induced by different processes. Recently, Piazzon *et al*. also found that carp IL-10a was more highly expressed under baseline conditions, and that IL-10b was up-regulated during an infection with spring viraemia of carp (SVCV)^29^. This is similar to our findings with CyHV-3, except that we also saw an up-regulation of IL-10a during infection. Moreover, Piazzon *et al*. reported that the two IL-10 homologues have a similar structure and exert similar bioactivity on phagocytes^29^. They suggested that the duplicates have undergone sub-functionalization, and are expressed differently due to differences in their promoter regions. Future studies focusing on further clarifying the nature of this divergence would be particularly interesting because IL-10 is an important regulator of broader immune responses, as previously discussed^22^,^51^,^52^. In another study that examined IL-10 homologue expression, Benedicenti *et al*. found that the homologues were expressed similarly in Atlantic salmon with amoebic gill disease, although they did not examine different phases of infection^51^. At a nucleotide level, modifications of the host IL-10a homologue sequence have been associated with greater resistance of carp to CyHV-3^22^. In this study, however, all of the wild Australian fish had identical IL-10 homologue sequences, suggesting that greater resistance due to SNP diversity in IL-10a^22^ may not affect this population. We suggest, however, that there may be selection for rare SNPs that confer resistance if CyHV-3 were to be released in Australia. These results highlight the need for greater study into homologue functionality of carp genes, including their influence on resistance and immune function. Moreover, studies examining immune responses in carp using PCR based techniques should be aware of the presence of homologues and design primers accordingly.

A successful humoral immune response relies on the production of a large diversity of B-cells with numerous receptors that are capable of matching a correspondingly large variety of antigen epitopes. The subsequent production of specific Ig can then be detected using serological techniques. In fish, however, the humoral response is relatively primitive, which can result in non-specific Ig production, longer response times, and variable levels of Ig production^26^,^27^. Consequently, often populations of carp, rather than individuals, are required for detection of CyHV-3-specific antibodies with ELISA-based protocols^26^. Moreover, cross-reactivity between CyHV-3 and CyHV-1 using ELISA can be problematic^21^. A potential alternative to these serological techniques is the use of high-throughput sequencing to assemble individual Ig transcripts, and then to identify the specific transcripts of interest. This approach may be more sensitive and specific, as well as providing the nucleotide sequence and information on gene and isoform expression^53^. This technique is relatively novel, particularly for fish, although it has been used to examine the antibody repertoire in healthy zebrafish^54^,^55^.

Here we used high throughput sequencing in fish to follow the changes in Ig repertoire during different phases of CyHV-3 infection. We found a highly reproducible pattern of Ig genotypes expressed by carp with no, or low levels of, CyHV-3 replication, i.e., all mock infected fish and fish in the persistent phase. In contrast, fish in the acute and reactivation phases produced a significantly greater diversity of Ig genotypes, suggesting the development of an adaptive immune response. Moreover, Ig transcripts from the acute and reactivation phases were under higher selection pressure in complementarity determining regions (CDR) and lower selection pressure in framework regions (FR), suggesting that clonal selection of high-affinity B-cells was occurring in these fish^56^,^57^. This can be inferred because clonal selection is manifested through fewer changes in regions that maintain structural integrity, i.e., FRs, but more changes in antigen recognition sites, i.e., CDRs^56^,^57^. Moreover, carp that were infected with CyHV-3 for the longest time, i.e., in the reactivation phase, had the highest overall selection pressure in CDR regions, further suggesting a clonal B-cell selection process. Only a small number of other studies have examined the selection pressure dynamics on fish Ig molecules, and these have produced inconsistent results^27^. For example, Yang *et al*. did not find any difference in selection pressure on catfish FRs and CDRs and suggested that clonal selection was inefficient in bony fish^58^. On the other hand, Magadán-Mompó *et al*. found higher frequencies of mutations in CDRs compared to FRs in medaka^59^, and this has also been seen in zebrafish^60^ and Nile tilapia^61^. Overall, our results indicate that Ig repertoire diversification was followed by clonal selection of high-affinity B-cells in carp infected with CyHV-3. In this experiment, however, only fish that succumbed to CyHV-3 infection were sampled and, thus, we could not detect a successful immune response or associated antibodies. Future studies will use carp that survive infection with CyHV-3 in order to detect CyHV-3-specific antibodies.

In summary, carp acutely infected with CyHV-3 altered the expression of thousands of genes, which were often involved in typical innate or adaptive immune response, or detoxification pathways. Interestingly, the carp predominantly mounted a humoral immune response, despite the probable establishment of CyHV-3 in cells. An intriguing possible explanation is that the virus itself has evolved to tip the balance of the host away from a cell-mediated response, and toward a humoral response, through the expression of a captured IL-10 homologue, thereby favouring virus survival. Moreover, many immune-related carp genes were found to be duplicated, and many of these showed dissociation of expression. Of particular interest were two IL-10 homologues that were expressed differently across different phases of infection, suggesting neo- or sub-functionalization. Finally, the carp Ig repertoire was examined during different phases of CyHV-3 infection. Ig diversification was detected during acute and reactivation phases, and this was followed by the selection of high-affinity B-cells, presumably signifying an adaptive humoral immune response.

## Methods

### Ethics statement

This study was carried out at the microbiologically-secure CSIRO-Australian Animal Health Laboratory (CSIRO-AAHL). All experiments were approved by the CSIRO-AAHL Animal Ethics Committee and performed according to the relevant guidelines and regulations.

### Experimental conditions and virus infection

Samples for this experiment were obtained from a previous study^62^, which includes histopathological data and detailed information on the experimental model. Briefly, juvenile common carp (mean length 12.1 ± 1.0 cm), *Cyprinus carpio,* were obtained from wild Australian stocks and acclimatized to laboratory conditions for 8 days. The carp were subjected to a 12 h light/12 h dark cycle and were given a commercial fish feed at 1% of their bodyweight per day. For these experiments, we chose 3 different phases of CyHV-3 infection in the fish: “acute”, “persistent” and “reactivation”. The acute group contained fish in the initial phase of active CyHV-3 infection at a permissive temperature; the persistent group included infected fish held at a low, non-permissive temperature for CyHV-3 replication; and reactivation fish were obtained by returning infected fish from the non-permissive temperature to a permissive temperature, thereby inducing an active infection. To achieve the desired groups, 60 carp were infected with 100 TCID_50_ ml^−1^ of an Indonesian CyHV-3 isolate (C07^63^) by immersion for 2 h at 22 °C. For the acute phase of infection, thirty of the infected fish were kept at 22 °C and individual fish were sacrificed as they became moribund. The remaining thirty infected fish were used to obtain the persistent and reactivation groups. They were initially held at 22 °C for 24 h to allow establishment of the CyHV-3 infection, and the infection was subsequently arrested by decreasing the water temperature to 11 °C over a period of 4 days at a rate of 2 to 3 °C per day. After 28 days at the non-permissive temperature, multiple fish were sacrificed for the persistent group. The virus was reactivated in the remaining fish by increasing the water temperature to 22 °C, again over 4 days, at a rate of 2 to 3 °C per day, and fish were sampled when moribund for the reactivation group. In addition to these groups, sixty mock-infected control carp were subjected to the same temperature regimes as the infected fish. The anterior kidney was dissected from sacrificed fish in each of the groups, and frozen at −20 °C in RNAlater (Ambion). After completion of the experiment, total RNA was extracted from the kidney of each fish with the AllPrep DNA/RNA extraction kit (Qiagen) following the manufacturer’s instructions.

### RNA-Seq and qRT-PCR

Three fish in each of the acute, persistent and reactivation phases, plus three randomly chosen mock fish, were selected for RNA sequencing (RNA-Seq). The RNA samples were sent to the Australian Genome Research Facility (AGRF, Melbourne, Australia), where messenger RNA (mRNA) was enriched in each sample by selection of polyA + tailed mRNA, which was then sequenced using two 150 bp single-end HiSeq lanes (Illumina). The raw RNA-Seq reads are available in the NCBI Sequence Read Archive under BioProject accession PRJNA314552. After our initial data analysis, one of the mock replicates was identified as a hybrid between common carp and goldfish (*Carassius auratus*) and was consequently removed from further analysis.

For validation purposes, the expression of several CyHV-3 open reading frames from the same samples used for RNA-Seq were determined using quantitative reverse transcription-PCR (qRT-PCR; for primers see Table 1 in Sunarto *et al*.)^62^. Expression from the open reading frames was normalised to expression of carp 18S rRNA to enable sample comparison. Duplicate reactions for each gene and each sample contained 12.5 μl of TaqMan One-Step RT-PCR Master Mix, 1 μl of RT-PCR Enzyme Mix (Applied Biosystems), 1.25 μl each of probe (5 μM) and forward and reverse primers (2 μM for 18S rRNA primers; 18 μM for viral primers), 2 μl of template RNA (100 ng per reaction), plus 5.75 μl of RNase-free water. The reactions were then amplified using a 7500 Fast Real-time PCR System (Applied Biosystems) with the following conditions: 48 °C for 30 min; 95 °C for 10 min; then 40 cycles of 95 °C for 15 s; 60 °C for 1 min. The results were analysed using 7500 software v.2.3 (Applied Biosystems).

### Data analysis

RNA-Seq reads were cleaned by removing Illumina adapters, trimming sequences with a quality score below 20 and removing reads with fewer than 75 bp with Trimmomatic v.0.33^64^. Any remaining ribosomal RNA was then removed from the reads using SortMeRNA v.2.0^65^ against the SILVA v.119 ribosomal database^66^. The high quality mRNA reads were mapped to the *Cyprinus carpio* genome v.1.0^30^ (available here: www.carpbase.org) and the CyHV-3 genome^9^ (GenBank accession DQ657948.1) using TopHat v.2.1.0^67^. Successfully mapped reads were assembled into separate transcriptomes for each individual sample using Cufflinks v.2.2.1, before being merged into a master assembly with Cuffmerge v.1.0.0, as recommended^68^. Counts for each gene were extracted from the mapping files using HTSeq v.0.6.0^69^ with the “union” mode for handling overlapping reads. The raw counts were used for differential expression testing in DESeq2 v.1.6.3, using a significance value of 0.05 and Benjamini & Hochberg corrections for multiple comparisons, as recommended^70^. Reads per kilobase of transcript per million mapped reads (RPKM) values were calculated using the counts from HTSeq v.0.6.0^69^ in Python v.2.7.11, and normalised log transformations of count data were conducted using DESeq2 v.1.6.3^70^. CummeRbund v.2.8.2^71^ was used to create dendrogram and PCA plots of gene expression similarities between replicates, and matplotlib-venn v.0.11.4 and Seaborn v.0.6.0^72^ were used to plot Venn diagrams and bar graphs. Significant pairwise CyHV-3 gene expression differences and the CyHV-3 expression heatmap were plotted using ggplot2 v.2.1.0^73^, and heatmaps of differentially expressed carp immune genes were constructed using pheatmap v.1.0.8. Samtools depth v.1.3^74^ was used on each of the bam alignment files to extract coverage over the CyHV-3 genome and carp interleukin-10 homologues, which was then normalised to the total number of mapped reads per million in Python v.2.7.11 and plotted using Gviz v.1.14.2^75^. Duplicated carp genes were detected by clustering high quality proteins from the carp genome (greater than 10 amino acids in length and less than 20% stop codons) using orthoMCL^76^, and extracting immune-related homologues with reciprocal best match results using BLASTp v.2.2.31^77^ with an e-value of 10^−5^.

### Detection of immunoglobulin repertoire

Immunoglobulin (Ig) diversity is generated through somatic recombination and hypermutation of variable regions, which means that Ig diversity cannot be adequately obtained by mapping RNA-Seq reads to a sequenced genome^53^,^56^. This can be overcome by independently assembling RNA-Seq reads into transcriptomes and identifying Ig transcripts in the resulting assembly^53^. To achieve this, the cleaned mRNA reads from above were re-assembled into separate transcriptomes in a genome-independent manner using Trinity v.2.0.6^78^, with a minimum contig length of 200 and a minimum k-mer coverage of 2. Transcripts with homology to Ig sequences were identified in the assemblies using BLASTn v.2.2.31^77^ with an e-value of 10^−3^ against the LIGM-DB database v.1.2.1^79^, which includes *Cyprinus carpio* sequences. IgBLAST v.1.4.0^80^ was then used on the matching transcripts to identify variable (V), joining (J) and diversity (D) regions, and to determine if they were in-frame and functional. The results were loaded into a Change-O v.0.3.2^81^ database and functional transcripts were assigned into clonal groups (defined as having the same V assignment, J assignment and J length) using *DefineClones.py* with the “m1n” substitution model and a distance of 7 for calculating distances between the sequences. The model and parameters were selected after visualisation of distance to nearest neighbour plots^81^. Testing for significant differences in Ig diversity between the infection phases was conducted using *testDiversity* in Alakazam v.0.2.3^81^ with 1000 bootstrap replicates. The selection pressure on Ig transcripts was estimated using observed and expected mutations and the Bayesian estimation of Antigen-driven SELectIoN (BASELINe) algorithm^57^. To examine positions of diversity within the V region, a global alignment of Ig transcripts was performed using clustalW^82^, and the Shannon entropy of each position was calculated using Python v.2.7.12 and plotted with Pandas v.0.17.1^83^.

### Amplification and sequencing of IL-10 homologues

PCR was used to confirm the presence of two interleukin-10 (IL-10) homologues in the carp genome. The first homologue (IL-10b; cycg006102) was amplified using the primers IL-10F: CGCCAGCATAAAGAACTCGT and IL-10R: TGCCAAATACTGCTCGATGT^13^. For the second homologue (IL-10a; cycg035130), the primers IL-10_H2F: CTAGGAGAGCAGCAAACCAGT and IL-10_H2R: TGCCAAATACTGCTCAATGT were designed based on an alignment of homologues in the carp genome assembly^30^. For each primer set, PCRs were compiled with 12.5 μl of HotStarTaq master mix (Qiagen, Hilden, Germany), 0.5 μl of the forward and reverse primer (18 μM), 9.5 μl of water and 2 μl of template DNA. The conditions for both PCRs were 95 °C for 15 min; then 40 cycles of 95 °C for 30 sec, 50 °C for 30 sec, 72 °C for 30 sec; and a final elongation step of 72 °C for 7 min. Amplified products were purified using the Wizard SV Gel and PCR Clean-Up Kit (Promega, Madison, USA) and sequenced in both directions using a Thermofisher 3500xl Genetic Analyser.

## Additional Information

**How to cite this article**: Neave, M. J. *et al*. Transcriptomic analysis of common carp anterior kidney during *Cyprinid herpesvirus 3* infection: Immunoglobulin repertoire and homologue functional divergence. *Sci. Rep.* **7**, 41531; doi: 10.1038/srep41531 (2017).

**Publisher's note:** Springer Nature remains neutral with regard to jurisdictional claims in published maps and institutional affiliations.

## Supplementary Material

Supplementary Information

## Figures and Tables

**Table 1:** Read quality and mapping results for the RNA-Seq libraries.

Sample	NCBI BioSample	Treatment	Total reads	Cleaned reads	Mapped to *Cyprinus carpio* genome	Mapped to CyHV-3 genome
1	SAMN04537393	Acute	28,645,158	25,048,054	18,741,223 (74.8%)	360,418 (1.4389%)
2	SAMN04537394	Acute	31,309,358	28,452,933	21,568,752 (75.8%)	5,434 (0.0191%)
3	SAMN04537395	Acute	33,802,985	30,992,654	23,530,831 (75.9%)	5,337 (0.0172%)
4	SAMN04537396	Persistent	33,014,462	30,415,615	23,004,950 (75.6%)	96 (0.0003%)
5	SAMN04537397	Persistent	32,699,016	30,139,958	22,157,303 (73.5%)	101 (0.0003%)
6	SAMN04537398	Persistent	31,940,121	29,333,923	22,088,110 (75.3%)	66 (0.0002%)
7	SAMN04537399	Reactivated	33,243,586	30,521,918	22,959,764 (75.2%)	407 (0.0013%)
8	SAMN04537400	Reactivated	33,933,720	31,064,624	23,427,770 (75.4%)	261 (0.0008%)
9	SAMN04537401	Reactivated	32,050,049	28,934,613	21,425,596 (74.0%)	9,059 (0.0313%)
10	SAMN04537403	Mock	31,401,721	28,838,999	20,698,717 (71.8%)	95 (0.0003%)
11	SAMN04537404	Mock	36,074,985	32,918,417	24,248,317 (73.7%)	39 (0.0001%)

^*^Cyprinus carpio genome is from www.carpbase.org and the CyHV-3 genome is GenBank #DQ657948.1.

**Table 2:** Top 10 most differentially expressed carp genes in each phase of CyHV-3 infection compared to the mock controls.

Gene ID	Function	Fold change (log_2_)	Adjusted p-value
*Acute compared to Mock*			
cycg013101	Nitric oxide synthase, inducible	6.9	4.9^−36^
cycg029192	Microfibril-associated glycoprotein 4	6.4	4.9^−24^
cycg038910	Solute carrier family 2	−4.9	5.9^−24^
cycg049360	Latexin	3.9	7.5^−24^
cycg047504	Cytoglobin 1	5.9	3.2^−23^
cycg025164	Myeloperoxidase	6.2	3.1^−22^
cycg005547	Glutathione S-transferase 3	−4.5	4.1^−22^
cycg018221	Signal peptide	6.3	5.7^−22^
cycg036267	Immune-responsive gene 1	6.6	1.2^−21^
cycg049796	Glutamate [NMDA] receptor-associated protein 1	4.3	2.8^−21^
*Persistent compared to Mock*			
cycg002335	Insulin-like growth factor-binding protein	−5.4	4.4^−18^
cycg027385	Single Ig IL-1-related receptor	−2.3	4.4^−18^
cycg041948	Cytosolic sulfotransferase 3	−3.7	5.1^−17^
cycg003666	Signal peptide	−6.1	5.2^−17^
cycg010925	Trans-1,2-dihydrobenzene-1,2-diol dehydrogenase	−4.2	1.0^−16^
cycg043850	Thymidine phosphorylase	5.1	5.0^−15^
cycg022138	Natterin-4	−5.6	9.2^−15^
cycg008033	Transmembrane protein C19orf77	−2.6	4.3^−14^
cycg046066	Sulfate transporter	−3.3	8.4^−14^
cycg041592	Cocaine- and amphetamine-regulated transcript protein	4.9	6.3^−13^
*Reactivated compared to Mock*			
cycg041089	Sodium- and chloride-dependent creatine transporter 1	5.3	3.8^−19^
cycg008033	Transmembrane protein C19orf77	−3.0	8.0^−18^
cycg011420	S-adenosylmethionine synthase isoform type-1	−5.1	3.2^−15^
cycg038910	Solute carrier family 2	−3.8	2.4^−14^
cycg002359	Myosin-11	−2.8	1.2^−12^
cycg003666	Signal peptide	−4.6	6.5^−12^
cycg042156	UPI0001DE912A related cluster	−2.8	6.5^−12^
cycg031384	CXC chemokine receptor type 7	−3.0	7.2^−12^
cycg005547	Glutathione S-transferase 3	−3.2	1.2^−11^
cycg030806	Growth/differentiation factor 15	3.6	1.6^−11^
