# Supplementary material for: Transcriptomic analysis of common carp anterior kidney during Cyprinid herpesvirus 3 infection: Immunoglobulin repertoire and homologue functional divergence
Source: Sci Rep. 2017 Feb 2;7:41531. doi: 10.1038/srep41531 (PMC5288646; doi:10.1038/srep41531)
Supplement: Supplementary Information [file srep41531-s1.doc]

**Supplementary Materials.**

Transcriptomic analysis of common carp anterior kidney during *Cyprinid herpesvirus* 3 infection: Immunoglobulin repertoire and homologue functional divergence

Matthew J. Neave, Agus Sunarto, Kenneth A. McColl

Contents:

Supplementary Figure S1

Supplementary Figure S2

Supplementary Figure S3

Supplementary Figure S4


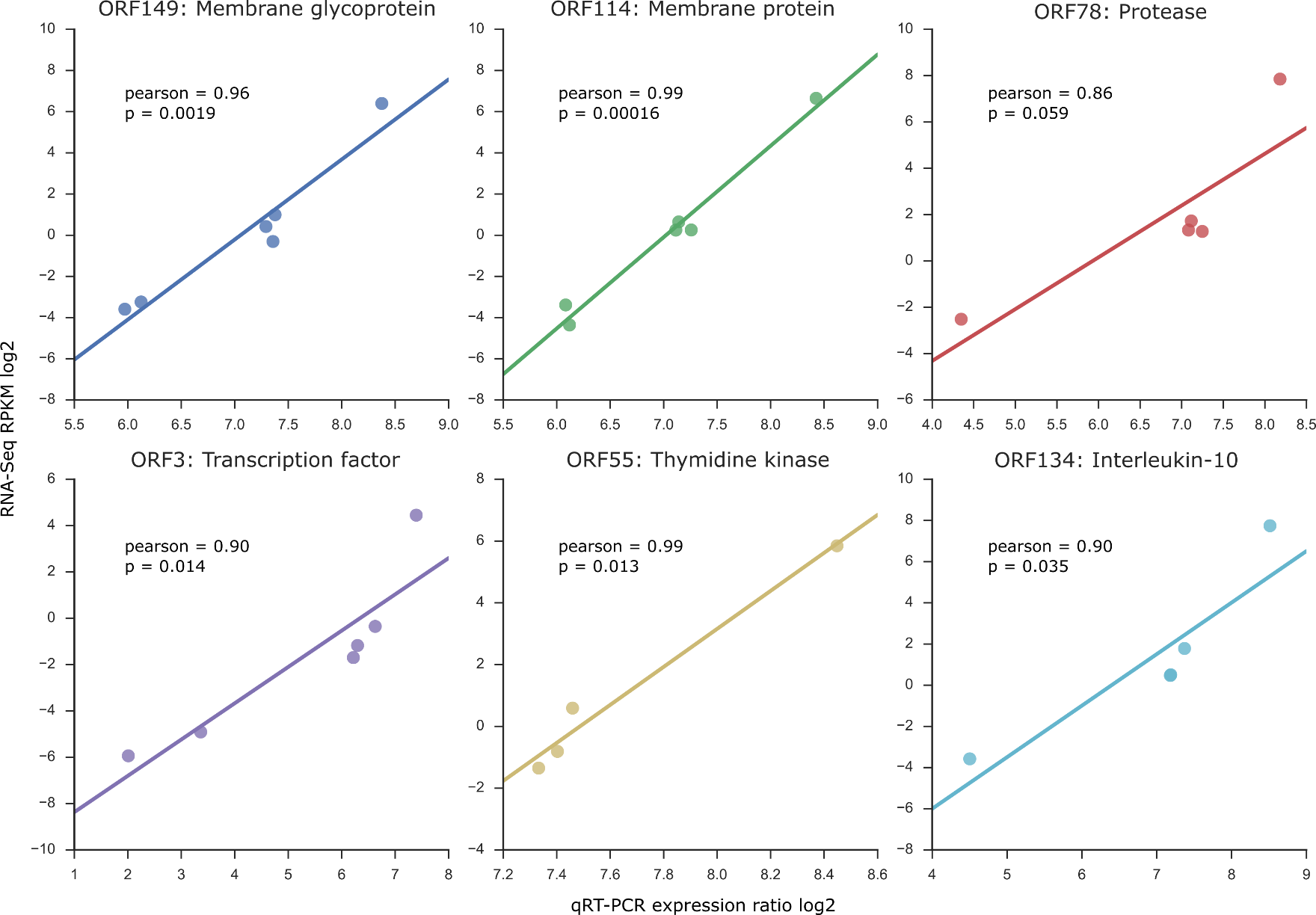


**Supplementary Figure S1.** Correlation of gene expression values determined by RNA-Seq and qRT-PCR. Six representative CyHV-3 genes were selected for comparison of the techniques. RNA-Seq values were normalised to reads per kilobase of transcript per million mapped reads (RPKM) and log_2_ transformed, and qRT-PCR values were normalized to carp 18S rRNA copy number and log_2_ transformed. Comparisons are only shown if detected using both techniques.


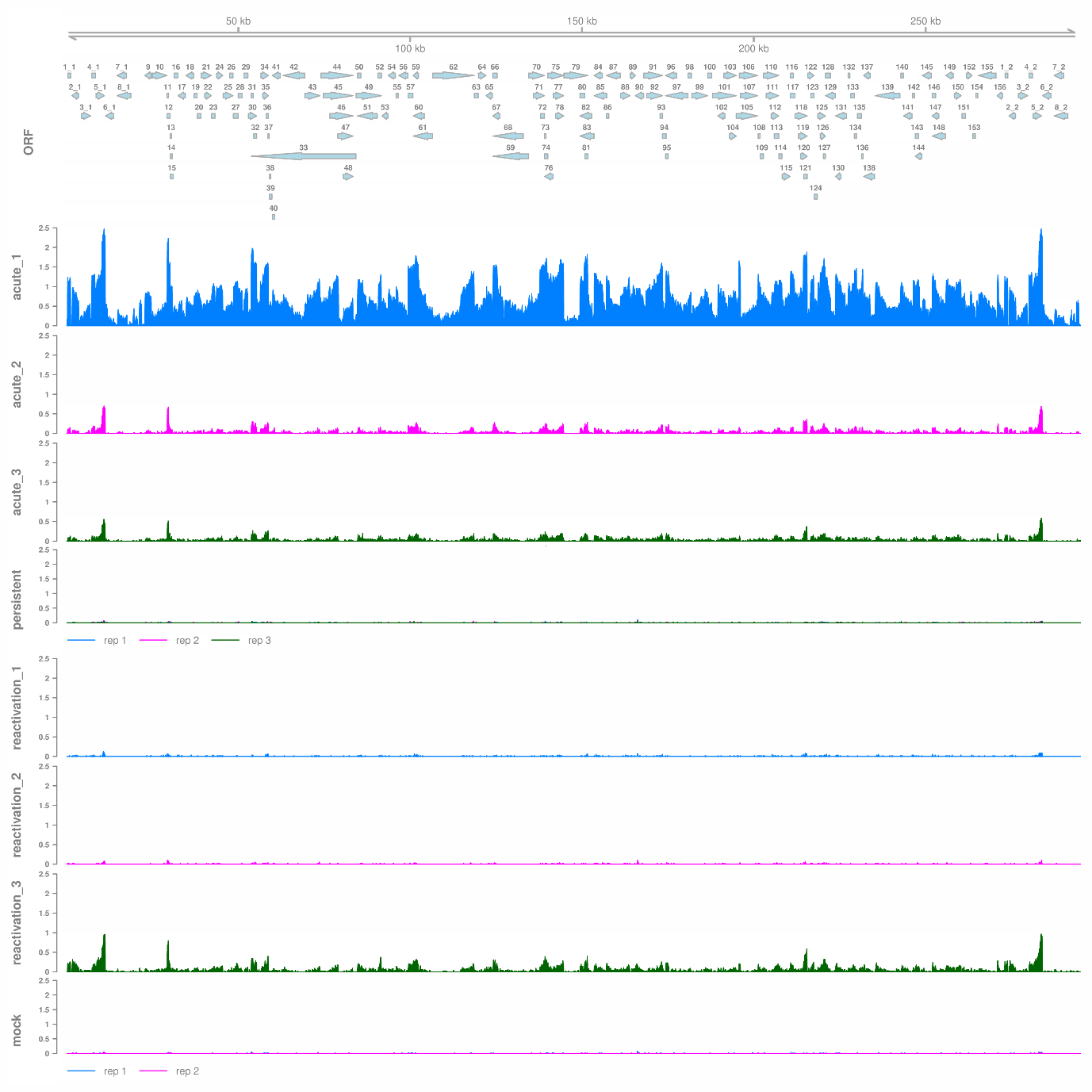


**Supplementary Figure S2.** Coverage plot of RNA-Seq reads over the CyHV-3 genome. The scales of each track were normalized to coverage per million total mapped reads (carp plus CyHV-3) and log_10_ transformed to enable comparison of the samples. The persistent and mock samples had very low coverage and the replicates from these groups were combined into a single track. The CyHV-3 genome and open reading frame (ORF) positions are from GenBank sequence DQ657948.1.


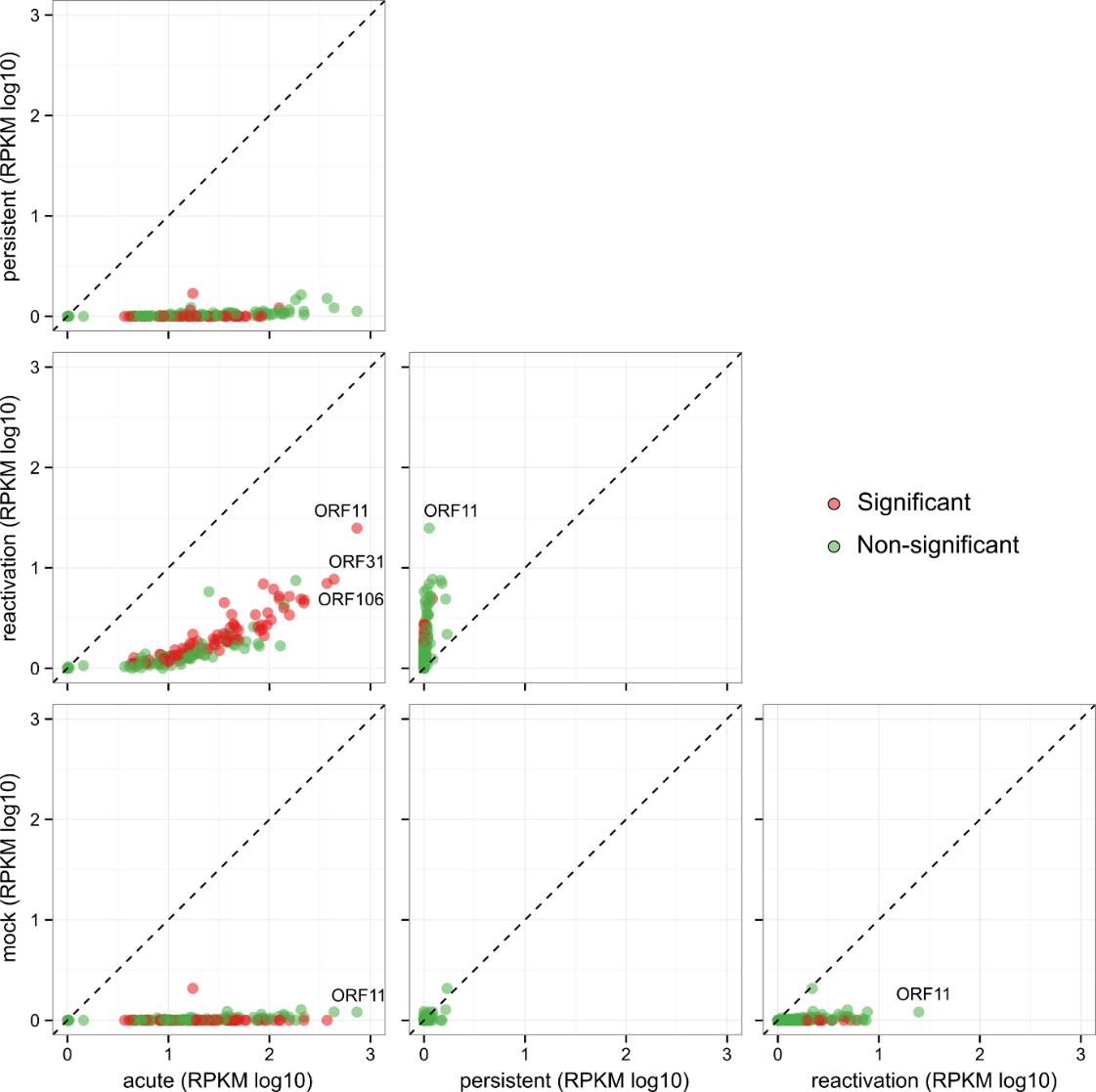


**Supplementary Figure S3.** Pairwise comparisons of CyHV-3 gene expression across the treatment types showing significantly differently expressed ORFs for each comparison. For some comparisons the CyHV-3 ORFs appear to have much higher expression but were found to be non-significant, which is likely due to variability within the replicates and a reduction in the power of the test.

**
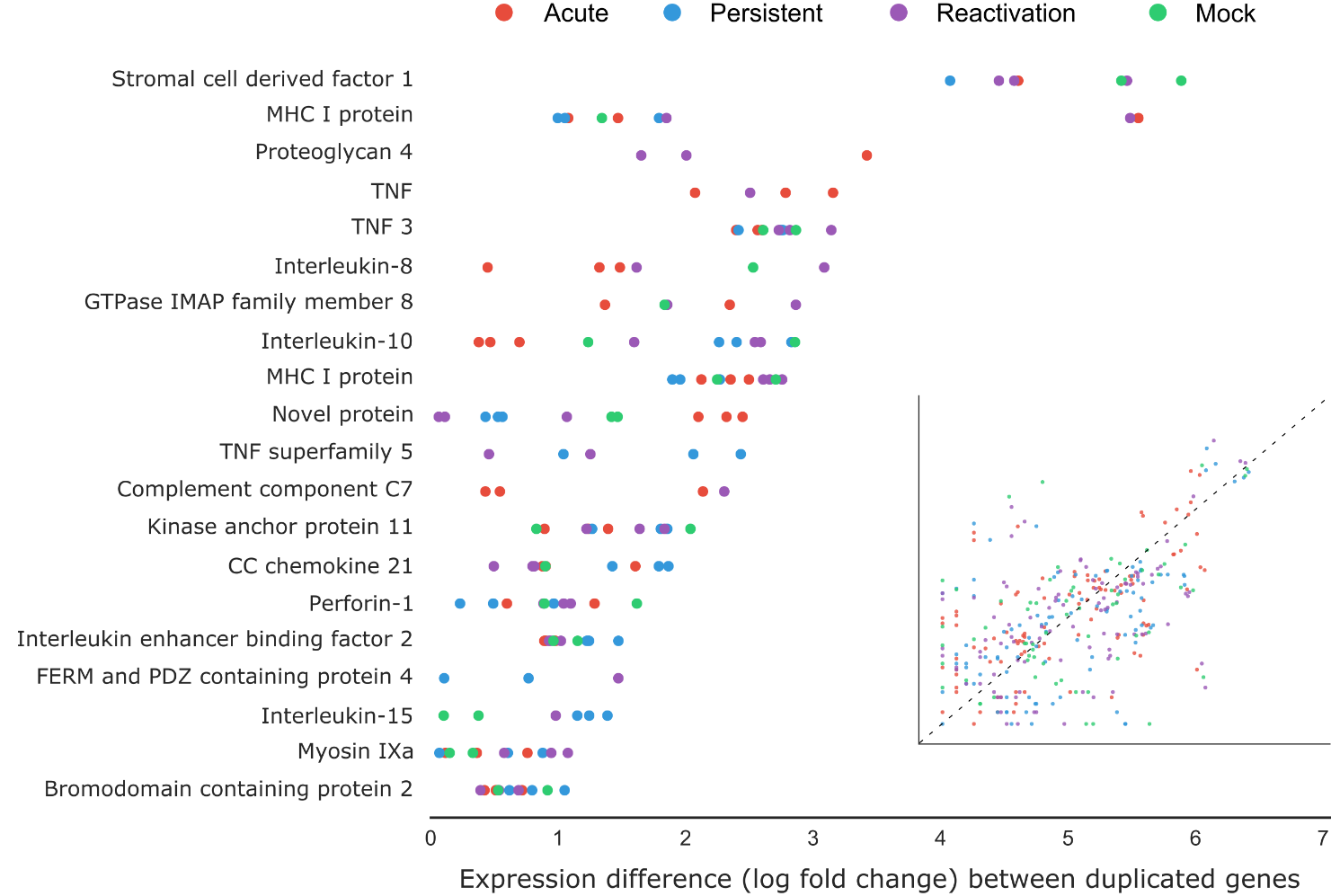
**

**Supplementary Figure S4.** Largest expression differences between duplicated immune genes in the carp genome and (inset) expression correlation between all duplicated immune gene.
